# Lansoprazole interferes with fungal respiration and acts synergistically with amphotericin B against multidrug-resistant *Candida auris*

**DOI:** 10.1080/22221751.2024.2322649

**Published:** 2024-03-03

**Authors:** Ehab A. Salama, Yehia Elgammal, Aruna Wijeratne, Nadia A. Lanman, Sagar M. Utturkar, Atena Farhangian, Jianing Li, Brigitte Meunier, Tony R. Hazbun, Mohamed N. Seleem

**Affiliations:** a Department of Biomedical Sciences and Pathobiology, Virginia-Maryland College of Veterinary Medicine, Virginia Polytechnic Institute and State University, Blacksburg, Virginia, USA; b Center for One Health Research, Virginia Polytechnic Institute and State University, Blacksburg, Virginia, USA; c Department of Biochemistry and Molecular Biology, Indiana University School of Medicine, Indianapolis, Indiana, USA; d Purdue Institute for Cancer Research, Purdue University, West Lafayette, Indiana, USA; e Department of Comparative Pathobiology, Purdue University, West Lafayette, Indiana, USA; f Department of Medicinal Chemistry and Molecular Pharmacology, Purdue University, West Lafayette, IN, USA; g Institute for Integrative Biology of the Cell (I2BC), Université Paris-Saclay, CEA, CNRS, Gif-sur-Yvette, France

**Keywords:** Lansoprazole, metabolites, PISA analysis, *C. auris*, fungal respiration, cytochrome *bc_1_*, *In vivo* mouse model

## Abstract

*Candida auris* has emerged as a problematic fungal pathogen associated with high morbidity and mortality. Amphotericin B (AmB) is the most effective antifungal used to treat invasive fungal candidiasis, with resistance rarely observed among clinical isolates. However, *C. auris* possesses extraordinary resistant profiles against all available antifungal drugs, including AmB. In our pursuit of potential solutions, we screened a panel of 727 FDA-approved drugs. We identified the proton pump inhibitor lansoprazole (LNP) as a potent enhancer of AmB’s activity against *C. auris.* LNP also potentiates the antifungal activity of AmB against other medically important species of *Candida* and *Cryptococcus.* Our investigations into the mechanism of action unveiled that LNP metabolite(s) interact with a crucial target in the mitochondrial respiratory chain (complex III, known as cytochrome *bc_1_*). This interaction increases oxidative stress within fungal cells. Our results demonstrated the critical role of an active respiratory function in the antifungal activity of LNP. Most importantly, LNP restored the efficacy of AmB in an immunocompromised mouse model, resulting in a 1.7-log (∼98%) CFU reduction in the burden of *C. auris* in the kidneys. Our findings strongly advocate for a comprehensive evaluation of LNP as a cytochrome *bc_1_* inhibitor for combating drug-resistant *C. auris* infections.

## Introduction

The multidrug-resistant fungus *Candida auris* has emerged as a serious global health threat in the last decade [1,2]. *C. auris* tends to survive on surfaces for months, tolerate thermal and disinfectant stress, and is easily transmitted to immunocompromised patients within a hospital environment [3,4]. Due to its high rate of mortality, exceptional resistance to antifungals, and ability to be rapidly transmitted, *C. auris* is the only fungal pathogen to be categorized among the top five urgent microbial threats according to the United States Centers for Disease Control and Prevention (CDC) [1]. One of the major phenotypic distinctions between other *Candida* species and *C. auris* is its extraordinary resistance profile to antifungals [5–7]. *C. auris* infections frequently resist two classes of antifungal drugs, azoles and polyenes [8,9]. However, a minor but alarmingly growing resistance (1-7%) has been reported towards the third class (echinocandins) [10,11]. Recently, some *C. auris* isolates were classified as pan-drug-resistant, tolerating all available antifungal drugs [12,13]. The recent increase in clinical outbreaks of infection caused by *C. auris* necessitates the development of effective therapeutic approaches to overcome the shortage in the current arsenal of antifungals [14–16].

The utmost priority in the development of antifungal agents is to ensure both safety and efficacy of the new therapy [17]. Various strategies have been employed to tackle the challenge of antifungal resistance, including the development of novel antifungal drugs with unique mechanisms of action [17]. Combination therapy has proven to be an effective approach in overcoming antifungal drug resistance, significantly contributing to preserving the existing arsenal of antimicrobial agents, reducing treatment duration, and mitigating drug toxicity [18]. Additionally, efforts have been directed towards identifying novel targets pivotal in the cellular stress response in fungi. Furthermore, there is a renewed focus on addressing the alarming rise in the number of patients highly susceptible to fungal infections [19].

Though it has a high level of toxicity, amphotericin B (AmB) has been used as a powerful broad-spectrum fungicidal agent for more than 60 years [20]. Resistance to AmB is rare among *Candida* species and most fungal pathogens. However, *C. auris* is an exception because 30% of the circulating *C. auris* isolates are resistant to AmB [21]. Increasing the AmB dose to overcome resistance is not feasible because of potential toxicity, particularly renal insufficiency [22]. Combination therapy is an alternative approach to overcoming the growing microbial infection resistance [23,24]. Therefore, we utilized this strategy to screen 727 FDA-approved drugs against an AmB-resistant *C. auris* strain to identify drugs that can restore the antifungal activity of AmB. We identified the gastric proton pump inhibitor (PPI) lansoprazole (LNP) as a potential candidate with a unique mechanism of action. Proteome Integral Solubility Alteration (PISA) analysis, RNA-Seq, rescue assays and activity testing of a collection of fungal mutants were used to uncover the mechanism of synergy between AmB and LNP. The fungal mitochondrial cytochrome *bc_1_* (complex III) was identified as a potential target for LNP. The AmB/LNP therapy was recognized as potentially interfering with fungal respiration, leading to increased oxidative pressure. We also evaluated the efficacy of the AmB/LNP combination to treat a *C. auris* infection in a mouse model of disseminated *C. auris* infection. Our findings revealed a novel strategy to revert *C. auris’* resistance to AmB by targeting the fungal mitochondrial cytochrome *bc_1_*.

## Materials and methods

### Fungal isolates, media, chemicals, and tested drugs

A total of 34 fungal isolates were used in this study in addition to mutant strains (details of clinical and mutant strains used in this study are listed in the Supplementary material).

### Screening of FDA-approved drugs and identification of LNP

To identify potent enhancers of AmB activity, the NIH drug collections library containing 727 FDA-approved drugs and clinical molecules, were screened *in vitro* (at a final concentration of 16 µM/mL) against *C. auris* AR0390 in the presence of a sub-inhibitory concentration (0.125×MIC) of AmB in RPMI 1640 (MOPS supplemented) media. Fungal growth was assessed after 24 h by measuring the OD at 600 nm. Among all identified hits, LNP was selected for further investigation in this study due to its favourable safety and pharmacokinetic profile.

### *In vitro* interaction between AmB and LNP against fungal isolates

The MICs of AmB and LNP against yeast isolates were identified using the CLSI M27-A3 guidelines [25]. Briefly, 96-well plates containing drugs and tested fungi in RPMI were incubated at 35°C for 24 h for *Candida* isolates, 48 h for *Aspergillus* isolates or 72 h for *Cryptococcus* isolates. The MIC was determined visually for each tested concentration. To evaluate the *in vitro* effectiveness of the AmB/LNP combination against a variety of *Candida* species, microdilution checkerboard assays were utilized, and the ΣFICI was calculated and interpreted as previously described [26,27]. Briefly, AmB and LNP were serially diluted in RPMI and then tested (alone and combined) in a 96-well plates against fungal isolates. The reading for checkerboard assays were taken visually employing the MIC criteria for assessment.

### Time-kill assay

A time-kill assay was performed to evaluate the change in the growth kinetics of *C. auris* in response to the AmB/LNP combination, as previously described [28,29].

### PISA analysis and target identification

We performed the PISA assay with biological triplicates to identify LNP’s target in *C. auris*, as previously described [30]. Briefly, *C. auris* AR0390 cultures were grown to the logarithmic phase in YPD and were subsequently collected, washed, and adjusted to 1 × 10^8^ CFU/mL in PBS. Cells were treated with either LNP (64 µg/mL) or DMSO. Samples were frozen in liquid nitrogen. Four freezing and thawing cycles were applied to obtain cellular lysates in the presence of a protease inhibitor cocktail. For PISA experiments, the lysates were then heated to eight different temperatures between 40.3 and 60.7 °C (40.3, 44.3, 48.3, 50.3, 52.1, 54.8, 58.3, and 60.7 °C) for 3 mins followed by incubation at 25 °C for 5 mins. The samples were ultracentrifuged at 150,000×*g* for 30 min at 4 °C, and the supernatant was collected. Subsequently, the soluble protein solutions were then precipitated, reconstituted in 8 M Urea, reduced/alkylated, digested using Trypsin/Lys-C, and labelled with TMT10 (Thermo Fisher Scientific Inc, MA, USA) as per vendor protocols for subsequent high-pH fractions and quantitative LC–MS/MS proteomics analysis. In the case of the global proteomics experiments, the same sample preparation procedure for LC-MS/MS was executed for 30 µg of total protein, excluding heat treatment and mixing. The detailed methods were adaptations from the previously reported [31].

## Transcriptomic analysis for drug-treated *C. auris*

### RNA extraction

Exponential *C. auris* AR0390 in RPMI 1640 medium were treated with DMSO (control), AmB (0.5 µg/mL), LNP (30 µg/mL), or the combination of AmB/LNP in triplicates for 3 hr at 35 °C. *Candida* cells were collected and washed three times with PBS, and the total RNA was extracted using a RiboPure^TM^-Yeast Kit (AmBion, AM1926, MA, USA) per the manufacturer’s instructions.

### Transcriptomic analyses and enrichment analyses of differentially expressed genes

150-bp paired-end DNA samples were sequenced using an Illumina NovaSeq6000. Fastp was used to quality-trim reads and eliminate Illumina TruSeq adaptor sequences [32]. Reads were aligned to the *C. auris* NCBI reference genome version B11221 using STAR aligner (v2.7.10a) [33]. The read counts mapped to each gene were calculated using FeatureCounts (v2.1) [34]. The EdgeR Bioconductor package (v3.16.5) was used to perform the differential expression analysis [35]. The Benjamini-Hochberg method was used to adjust the *P*-values for multiple testing. The cutoff for the significant differential expression was chosen at a FDR of 0.05. DEGs were annotated by blasting the sequences of identified DEGs versus *C. albicans* SC5314 Genome Database with an e-value cutoff of 0.01. Cluster Profiler Bioconductor package (v2.4.3) was used to perform Gene Ontology analysis of DEGs [36].

### ROS level measurement

As per the manufacturer's instructions, the cell-permeant H2DCFDA kit was utilized to quantify ROS levels in response to drug treatments. As earlier described [9], overnight grown *C. auris* AR0390 cells were collected, washed with PBS, and diluted to ∼1 × 10^7^ cells/mL in RPMI. The cells were treated with LNP (32 µg/mL), AmB (0.5 µg/mL), or a combination of both drugs for 3 hr at 35 °C. A fluorescent dye, 29,79-dichlorodihydrofluorescein diacetate (H2DCFDA), was added to a final concentration of 50 µM and incubated at 35 °C in the dark for 30 min. Cells were then collected by centrifugation at 3,000 rpm for 5 min and washed three times before being resuspended in PBS and transferred to opaque 96-well plates. The fluorescence signals were measured at an excitation/emission wavelength of 428/535 using a BioTek Synergy H1 microplate reader.

### *S. cerevisiae* growth assays

The following growth media were used: YPD (1% yeast extract, 2% peptone, 3% glucose) and YPEtOH (1% yeast extract, 2% peptone, 2% ethanol). Cultures were inoculated at an OD_600nm_ of 0.2 from freshly grown cultures on YPD plate and incubated at 28°C with vigorous shaking for up to several days. OD_600nm_ were measured at different times. The experiments were repeated at least twice, and the data was averaged.

### Complex III (cytochrome *bc*_1_) assay

Mitochondria were prepared from *S. cerevisiae* strain AD1-9 as described previously [37]. NADH-cytochrome *c* reductase activities were measured by monitoring the rate of cytochrome *c* reduction spectrophotometrically at 550-540 nm in a two-minute time course. Measurements were performed at room temperature in 1 mL of 10 mM potassium phosphate pH 7, 2 mM KCN and 20 μM cytochrome *c*. Mitochondria were added at 35 μg protein/mL. The reaction was initiated by adding 0.8 mM NADH, and the initial rate of cytochrome *c* reduction was recorded. The measurements were repeated twice, and the data was averaged. The data are presented as ΔA_550-540_/min.

### Molecular modelling

Molecular docking was performed using Glide v7.9. (Schrodinger, LLC), with two ligands (lansoprazole and lansoprazole sulfide), Complex III from *C. albicans* (PDB ID: 7RJA) and the bovine *bc1* with UHDBT (PDB ID: 1SQV). The ligands and proteins were prepared using Maestro (Schrodinger, LLC), and various protonation states of the ligands were considered. Both structures have corresponding binding sites that were identified by the SiteMap programme in Maestro. We employed the Glide Extra Precision (XP) docking module from Schrödinger Suite. XP employs a physics-based scoring function that accounts for van der Waals forces, electrostatic interactions, and solvation effects. It also incorporates ligand desolvation and receptor flexibility, enabling accurate prediction of ligand binding affinities and conformations [38]. The docking results were analyzed and visualized using Maestro and PyMOL.

### Evaluation of the *in vivo* efficacy in a murine model of *C. auris* infection

To assess the *in vivo* efficacy of the AmB/LNP combination, we utilized a previously established mouse model of disseminated *C. auris* infection [39–41]. Briefly, six-weeks-old female CD-1 mice were divided into groups (10 mice per group) and were rendered neutropenic by injecting 2 i.p. doses of cyclophosphamide 4 days (200 mg/kg) and 1 d (150 mg/kg) prior to the fungal challenge. On the day of infection, mice were infected with 4 × 10^7^/mouse of *C. auris* AR0390 suspended cells in PBS. Two hours later, infected mice were treated with the vehicle, AmB (0.5 mg/kg), LNP (300 mg/kg), or a combination of AmB/LNP. All treatments were given once daily (i.p. for AmB and orally for LNP) for two days. On day three, all mice were euthanized. The kidneys were extracted, homogenized, and plated onto YPD agar supplemented with chloramphenicol (75 µg/mL). The colony-forming units were recorded after 24 hr incubation at 35°C.

## Statistical analysis

The number of biological replicates is noted in each experiment, and the statistical analysis was carried out using a one-way ANOVA of Dunnett’s test for multiple comparisons using GraphPad Prism 8 (GraphPad Software, La Jolla, CA) unless otherwise noted.

## Results

### Drug library screen identifies LNP as a potentiator of AmB’s antifungal activity

**Figure 1.**
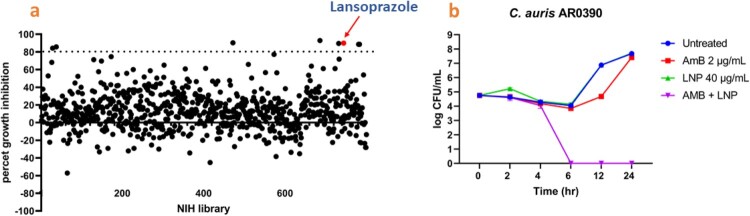
A screen of 727 FDA-approved drugs identified lansoprazole (LNP) as a potent potentiator of the antifungal activity of amphotericin B (AmB) against *C. auris*. a) An NIH drug collection library containing 727 drugs was screened at 16 µM in RPMI medium at 35 °C for 24 hr in the presence of 0.125×MIC of AmB (0.25 µg/mL). Growth of *C. auris* AR0390 was detected by measuring the optical density (OD_600_). The dotted line indicates 80% growth inhibition of *C. auris*. b) A time-kill assay of AmB at 2 µg/mL (1×MIC), LNP at 40 µg/mL, or a combination of the two drugs in RPMI media against *C. auris* AR0390 at 35°C for 24 hr. Time-kill assay results are shown as the mean values of CFU ± SD obtained from two independent experiments.

We screened the NIH Clinical Collections 1 and 2 of 727 FDA-approved drugs and clinical molecules previously used in human clinical trials with known safety profiles [42] to identify hits that enhanced the activity of AmB against *C. auris*. Two screens were conducted on a multidrug-resistant isolate of *C. auris* (AR0390) with a minimum inhibitory concentration [MIC] of AmB = 2 µg/mL ( = 2.2 µM/mL). One screen included a sub-inhibitory concentration of AmB (0.125×MIC) and 16 µM of the library, while the other screen omitted the use of AmB. In the presence of LNP and AmB, 90% inhibition of *C. auris* growth was observed. Thus, LNP was identified as a robust potentiator of the antifungal activity of AmB (Figure 1a).

### Synergistic interactions between AmB and LNP against *C. auris* and other fungal species

The *in vitro* interaction between AmB and LNP was investigated against a panel of 20 clinical isolates representing the four main clades of *C. auris*. Among these isolates, 11/20 (55%) were resistant to AmB (MIC = 2 µg/mL). Notably, LNP demonstrated a synergistic interaction with AmB in 90% (18 out of 20) of the isolates. The two isolates that did not exhibit synergy also showed a reduction in the MICs of AmB to the susceptible levels (Table 1). When we examined the AmB/LNP combination against other medically important *Candida* and *Cryptococcus* species (*C. albicans*, *C. glabrata* (now named *Nakaseomyces glabratus*), *C. tropicalis, C. parapsilosis*, *C. krusei* (now named *Pichia kudriavzevii*)*, C. gattii* and *C. neoformans*), AmB/LNP exhibited a synergistic relationship against tested isolates with fractional inhibitory concentration index (ΣFICI) values that ranged between 0.25 to 0.50 (Table S1). The results affirm the broad-spectrum synergistic interactions between AmB and LNP against a majority of significant fungal pathogens.

**Table 1.** Effect of the combination of amphotericin B with lansoprazole against 20 Candida auris isolates.

Isolate ID	Clade No.	MIC (µg/mL)				ΣFICI	Interpretation
		Alone		Combined			
		AmB	LNP	AmB	LNP		
***C. auris* 381**	II	1	128	0.25	16	0.38	SYN
***C. auris* 382**	I	1	>128	0.25	16	0.31	SYN
***C. auris* 383**	III	1	128	0.125	16	0.25	SYN
***C. auris* 384**	III	1	128	0.25	16	0.38	SYN
***C. auris* 385**	IV	2	>128	0.5	32	0.38	SYN
***C. auris* 386**	IV	2	128	0.5	16	0.38	SYN
***C. auris* 387**	I	1	128	0.25	32	0.50	SYN
***C. auris* 388**	I	2	128	1	16	0.63	IND
***C. auris* 389**	I	2	128	0.5	32	0.5	SYN
***C. auris* 390**	I	2	128	0.5	32	0.5	SYN
***C. auris* CBS 10913**	II	1	128	0.25	16	0.38	SYN
***C. auris* CBS 12372**	II	1	128	0.25	16	0.38	SYN
***C. auris* CBS 12373**	I	1	128	0.25	16	0.38	SYN
***C. auris* CBS 12766**	I	2	128	1	16	0.63	IND
***C. auris* CBS 12770**	I	2	>128	0.5	64	0.50	SYN
***C. auris* CBS 12771**	I	2	>128	0.5	32	0.38	SYN
***C. auris* CBS 12772**	I	2	>128	0.5	32	0.38	SYN
***C. auris* 1100**	–	2	128	0.5	16	0.38	SYN
***C. auris* 1101**	II	1	64	0.25	8	0.38	SYN
***C. auris* 931**	IV	2	256	0.5	16	0.31	SYN

**AmB**: amphotericin B, **LNP**: lansoprazole, **ΣFICI**: fractional inhibitory concentration index, **SYN**: synergy, **IND**: indifference.

### LNP restores AmB fungicidal properties in a time-kill assay

A time-kill assay was conducted to identify the killing kinetics of the AmB/LNP combination. LNP (40 μg/mL = 108 µM/mL) restored the fungicidal properties of AmB (2 μg/mL) against resistant *C. auris* within 6 hr. However, AmB alone did not prevent *C. auris* growth over 24 hr (Figure 1b). This outcome highlights the potential of the AmB/LNP combination to demonstrate rapid fungicidal activity, representing a notable advantage in antifungal therapy.

### Proteome Integral Solubility Alteration (PISA) analysis identifies mitochondrial cytochrome *bc1* (complex III) as a potential target

**Figure 2.**
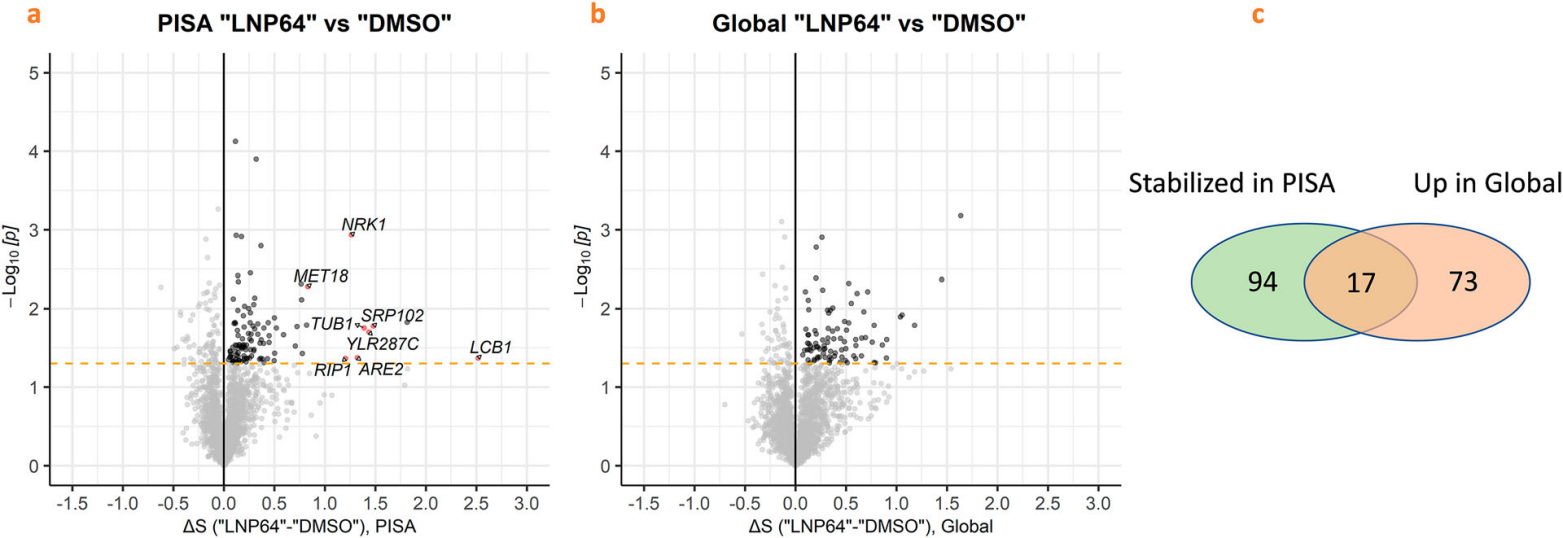
Volcano plots indicating “stabilized” (from PISA) and “up” regulated proteins (global proteomics) by LNP treatment compared to DMSO treatment. a) Corresponding volcano plot (ΔSm, p) of the PISA experiments for LNP-treated (64 µg/mL) vs. DMSO-treated *C. auris* AR0390 cells demonstrating the strong positive outliers (potential protein targets). b) Corresponding volcano plot (ΔSm, p) of the Global experiments for LNP-treated (64 µg/mL) vs. DMSO-treated *C. auris* AR0390 cells showing proteins increased in abundance in the Global experiment (Up in Global). Results are presented from triplicate experiments; black dots represent the proteins stabilized in the PISA Experiment, and the dotted line identifies the statistical significance (*p* < 0.05). c) Summary of the total number of proteins stabilized in PISA or up in the Global proteomic analysis. (All the protein IDs and relevant details can be visualized in the supplementary spreadsheet (Excel spreadsheet_S1).

The PISA method is an emerging mass spectrometry-based technique to identify protein-chemical interactions [43]. We employed the PISA method to identify potential fungal protein targets of LNP by directly treating *C. auris* with the drug. LNP interacting proteins were identified by analyzing the thermal shifts of individual proteins in the *C. auris* proteome after cells were treated with LNP (64 μg/mL) compared with DMSO-treated cells. The samples were also subjected to global proteomics experiments to measure overall protein abundance in response to LNP treatment. The thermal proteome profiling of the LNP-treated cells between 40.3 and 60.7 °C is listed in Table S2, and the supplemental data (Excel spreadsheet_S1). A total of 2631 proteins were identified between the PISA and global proteomics experiments. The ΔS_m_ distribution was calculated for each experiment, reflecting the solubility or abundance difference between the treated and untreated samples. Proteins with increased abundance were excluded, resulting in the identification of eight statistically significant proteins that exhibited increased solubility in the presence of LNP as represented by the ΔS_m_ distribution (Figure 2 and Table S2). We generated a catalog of *C. albicans* and *S. cerevisiae* orthologs to all the *C. auris* genes to facilitate gene ontology analysis and compensate for the poor annotation of the *C. auris* genome. Among these potential targets, we focused on mitochondrial complex III, also referred to as cytochrome *bc_1_*, because the complex was previously identified as a target of LNP in *Mycobacterium tuberculosis* [44]. B9J08_005505 is the *C. auris* ortholog of the Rieske protein, a member of cytochrome *bc_1_* complex, which is encoded by the *RIP1* gene in *C. albicans* and *S. cerevisiae*. Additionally, interference with the mitochondrial electron transport chain, which is involved in fungal respiration, significantly enhances AmB activity [45]. We note that cytochrome *b* and cytochrome *c_1_* interact with the Rieske protein to form the redox site of the complex, and so all three proteins may behave similarly in a PISA experiment. However, cytochrome *b* peptides were not identified in the proteomic experiments, so the PISA analysis could not assess stabilization by LNP treatment. In addition, cytochrome *c_1_* had a high ΔS_m_ value but was excluded as a PISA hit because it also had a high protein abundance value in the LNP-treated sample.

We also investigated tubulin as a target because a similar PPI, omeprazole, has been implicated in binding to structural proteins such as tubulin in *Giardia* [46]. Sensitivity studies using *S. cerevisiae* deletion strains sensitive to tubulin disruption suggest that tubulin is not a major target (Table S4). The other proteins identified by the PISA experiment may interact with LNP but might not be the primary target based on our validation studies demonstrating the defects in mitochondrial function upon exposure to LNP (Tables S3, S4 and S5).

### Transcriptomic analysis of *C. auris* treated with AmB/LNP or LNP suggests a cellular response to oxidative damage

To identify which biological pathways affected by the AmB/LNP treatment, we performed a comparative RNA-Seq analysis of *C. auris* AR0390 treated with DMSO, AmB (0.5 µg/mL), LNP (30 µg/mL), or the AmB/LNP combination which are subtoxic concentrations of compounds. Differentially expressed genes (DEGs) between the different treatment groups were identified, and genes with a False Discovery Rate (FDR) < 0.05 were marked as statistically significant. Massive transcriptome changes were observed for the samples treated with AmB and AmB/LNP, including 4010 and 4160 statistically significant DEGs, respectively, which is expected for the near subtoxic concentrations of AmB utilized. The AmB and cotreated sample of AmB/LNP, when compared to DMSO, demonstrated similar enrichment in DEGs associated with DNA replication and repair. However, comparing AmB/LNP to AmB revealed enrichment in cellular respiration, mitochondrial organization, and ion transport under cotreatment conditions (Table S5).

**Figure 3.**
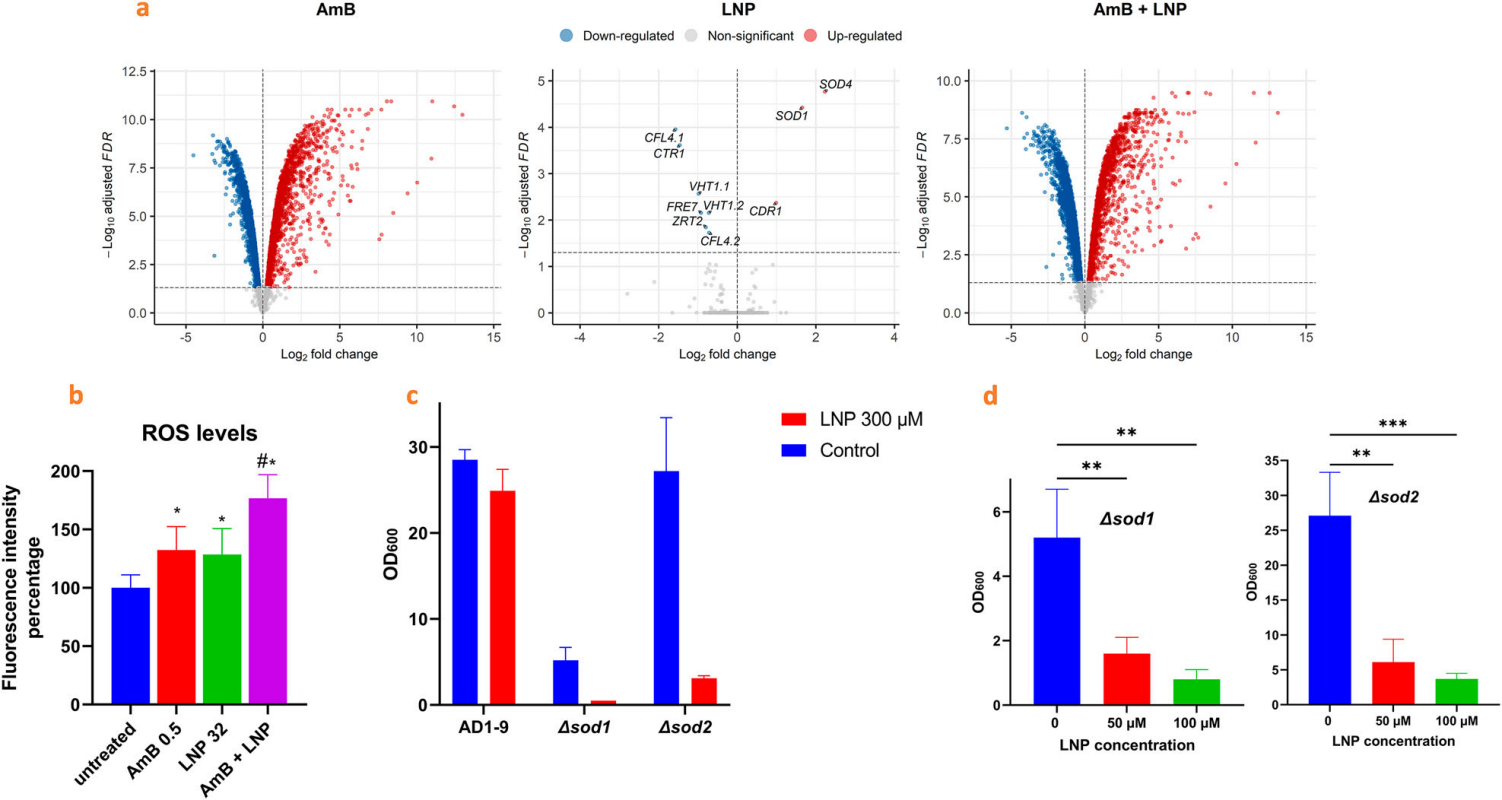
RNA-Seq analysis and oxidative stress response upon AmB/LNP treatment. a) Volcano plot highlights differentially expressed genes (DEGs) from *C. auris* AR0390 cells treated with AmB (0.5 µg/mL), LNP (30 µg/mL) or the AmB/LNP combination. The LNP-treated group (in the middle) showed three upregulated and 7 downregulated genes. Superoxide dismutase and oxidoreductase orthologs appeared among the up and downregulated genes. Genes with FDR < 0.05 were considered differentially expressed (4010, 10, and 4160 DEGs for samples receiving AmB, LNP, and AmB/LNP, respectively). Results are presented from three independent experiments. b) ROS level measurement. 1 × 10^7^ *C. auris* AR0390 cells/mL were treated with AmB (0.5 µg/mL), LNP (32 µg/mL), or a combination of both drugs for 3 hr. ROS levels were measured by incubating fungal cells with a cell-permeant H2DCFDA kit, and the fluorescence intensity was adjusted to the untreated and presented from two independent experiments. c) Comparison of LNP sensitivity between *S. cerevisiae* AD1-9 and two derived mutants (Δsod1 and Δsod2). The cells were grown in YPEtOH media with or without 300 μM LNP. The OD_600nm_ were recorded after 72 hr incubation. d) LNP dose-dependent sensitivity of the Δsod1 and Δsod2 mutants. The cells were cultured as in c).

Remarkably, only 10 DEGs (3 upregulated and seven downregulated) were detected in the LNP-treated group (Figure 3a). Two out of the three upregulated genes were found to be superoxide dismutase homologues (*SOD1* and *SOD4*) and the third gene was the multidrug transporter, *CDR1*. The common theme amongst the downregulated DEGs was metal ion uptake because the downregulated genes included orthologs to ferric reductases (*FRE1*, *FRE2* and *FRE7*), a copper ion transporter (*CTR1*) and a zinc ion transporter (*ZRT2*) (Figure 3a). Interestingly, two additional genes involved were orthologs of a *C. albicans* gene which is implicated as a biotin importer (*VHT1*). *C. auris* appears to have an expansion of the nicotinic acid transporter gene family (*TNA1* and *VHT1*) compared to *C. albicans* and *S. cerevisiae* [47]. In another study, a decrease in the expression of the VHT1 orthologs, namely B9J08_002974 and B9J08_004448, was noted when *C. auris* was cultivated on carbon sources other than glucose [47]. The LNP downregulated genes were enriched for GO terms involving metal ion transport (biological process), cell periphery (cellular component) and ferric-chelate reductase activity (molecular function). All significant up and downregulated genes are shown in Figure 3a. The downregulation of metal ion import and upregulation of SODs suggests a cellular response that attempts to counteract oxidative damage initiated by LNP. Consistent with the notion of LNP-mediated oxidative damage, the SODs are antioxidant enzymes that detoxify ROS [48], and metal ion homeostasis is crucial in minimizing ROS formation [49]. Interestingly, the GO enrichment analysis on differentially expressed genes from LNP-treated cells highlighted the mitochondrial intermembrane space as the top Cellular Component (CC) location (Fig. S3).

### LNP increases reactive oxygen species (ROS) levels and exhibits potent activities on SOD deletion mutants

Since mitochondria are a prime source of ROS, we investigated the ROS levels in response to drug treatments. We used the ROS indicator, 29,79-dichlorodihydrofluorescein diacetate (H2DCFDA), to quantify levels of ROS generated from *C. auris* cells treated with AmB, LNP or a combination of AmB/LNP. LNP and AmB treatments increased the generated ROS levels compared to untreated fungal cells by 29% and 32%, respectively. As expected, the AmB/LNP combination led to a 77% increase in ROS levels, significantly higher than fungal cells treated with either LNP or AmB alone (Figure 3b). To confirm the role of ROS in LNP activity, we measured the inhibitory effect of the drug on *S. cerevisiae* mutants lacking either the cytoplasmic or the mitochondrial superoxide dismutase, Sod1 and Sod2. Both mutants showed high sensitivity to LNP with reduced growth by 90% and 89%, respectively, as compared to the parent strain AD1-9 (Figure 3c). The sensitivity of Δ*sod1* and Δ*sod2* mutants to LNP proved dose-dependent (Figure 3d).

### LNP interferes with fungal respiratory function

**Figure 4.**
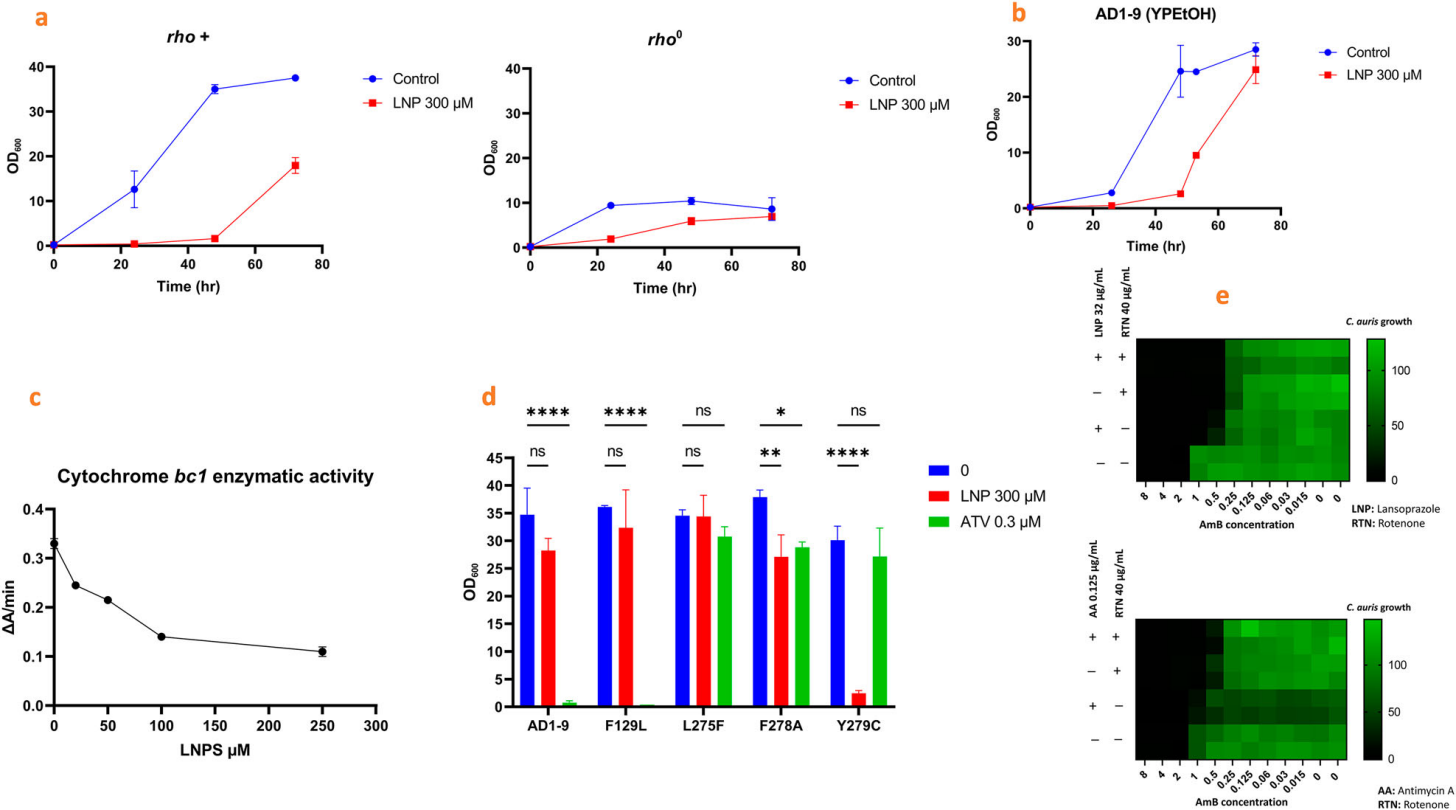
Lansoprazole (or its metabolites) interferes with the fungal mitochondrial respiration and cytochrome *bc*_1_ activity. a) Comparison of LNP sensitivity between AD1-9 (rho^+^) and its derivative lacking mtDNA, and thus a respiratory function (rho°). The cells were grown in YPD medium with or without 300 μM LNP. The OD_600nm_ were recorded at 24, 48 and 72 hr. b) Effect of LNP on the growth of *S. cerevisiae* AD1-9 on medium containing ethanol as a sole carbon source (YPEtOH) over 72 hr. c) Effect of LNP metabolite (LNPS) on cytochrome *bc*_1_ activity. Mitochondria were prepared from *S. cerevisiae* AD1-9 strain. The enzyme activity was measured by monitoring the rate of cytochrome *c* reduction spectrophotometrically at 550-540 nm using NADH as an electron donor in the presence of increasing concentration of LNPS. d) Effect of cytochrome *b* amino acid replacement on the susceptibility of *S. cerevisiae* to LNP. The cytochrome *b* mutants and their parent strain AD1-9 were grown 4 days in YPEtOH medium with or without 300 μM LNP or 0.3 μM atovaquone (ATV). Asterisks indicate a statistical significance * (*P* < 0.1), ** (*P* < 0.01), **** (*P* < 0.0001). e) Effect of the respiratory chain inhibitor, rotenone (targeting complex I), on the synergistic effect between AmB and both LNP and antimycin A (targeting complex III). The heat-maps represent *C. auris* AR0390 growth after 24 hr relative to the untreated control. Top panel, the effect of rotenone on the synergistic relationship between AmB and LNP; lower panel, the effect of rotenone on the synergistic relationship observed between AmB and AA.

To further confirm that fungal respiration is a potential target for LNP, we compared the activity of LNP on the growth of *S. cerevisiae* parent strain AD1-9 and its derived mutant rho° lacking mitochondrial DNA (mtDNA) and thus lacking a respiratory function. The cells were grown in a YPD medium with a vigorous culture agitation for high-level aeration. In these conditions, the parent strain (rho^+)^ uses mainly the respiratory function, while the rho° cells exclusively produce their energy by glycolysis. In parallel, we cultured the rho^+^ in rich media supplemented with ethanol as the carbon source instead of glucose (YPEtOH) so the cells exclusively use the respiratory function. At 48 hr, the rho^+^ (in YPD and YPEtOH) was inhibited by ∼95%, while the rho° was inhibited by 40% (Figure 4a and 4b). The decreased sensitivity of the rho° cells indicates that the respiratory function plays a role in LNP inhibitory activity. The effect started to fade after 72 hr potentially due to the instability of LNP in the culture media.

Similar results were observed for the *C. albicans* SC5314 strain growth in the presence of glucose versus glycerol as a carbon source (Fig. S1). Due to the inability of most *C. auris* isolates to grow on glycerol or ethanol media as the primary carbon source [50], we could not evaluate the impact of LNP on the growth of *C. auris* with both media.

### Cytochrome *bc_1_* is a target of LNP as shown by using cytochrome inhibitor rescue assays and cytochrome *b* mutations

If cytochrome *bc*_1_ is the target for LNP as revealed by PISA, we hypothesized that the well-known cytochrome *bc*_1_ inhibitor antimycin A could also potentiate the activity of AmB against *C. auris*. By the same reasoning, the complex I inhibitor rotenone, which blocks the electron transport chain earlier, should inhibit the synergistic relationship observed by either LNP or antimycin A in combination with AmB. As expected, antimycin A (0.125 µg/mL) showed similar potential as LNP to enhance the activity of AmB against *C. auris* AR0390. Our hypothesis was further supported when rotenone (40 µg/mL) negated the synergistic relationship of either LNP or antimycin A with AmB (Figure 4e).

We evaluated the effectiveness of LNP on four *S. cerevisiae* strains that harboured amino-acid substitutions in cytochrome *b*, namely F129L, L275F, F278A and Y279C. These mutants were previously generated using the mitochondrial transformation technique [51]. The mutated residues are located in the so-called Q_o_ site formed by cytochrome *b* interacting with the Rieske protein to create the ubiquinol oxidation site within the cytochrome *bc*_1_ complex. This site is the same target that a lansoprazole metabolite, lansoprazole sulfide, has been demonstrated to engage in *Mycobacterium tuberculosis* [44]. In parallel, we tested the sensitivity of these mutants to the well-known inhibitor atovaquone that binds in the Q_o_ site [52]. The parent strain and F129L were fully inhibited by atovaquone, while L275F, F278A and Y279C were resistant. Interestingly, Y279C showed a substantially increased sensitivity to LNP (Figure 4d), suggesting that a cysteine replacing the tyrosine at position 279 might facilitate the inhibition of the cytochrome *bc*_1_ by LNP or its metabolite(s).

We then tested whether LNP or its metabolite lansoprazole sulfide could directly inhibit yeast cytochrome *bc*_1_ activity. To that end, we prepared mitochondria from *S. cerevisiae* AD1-9 strain and measured the rate of cytochrome *c* reduction using NADH as an electron donor in the presence of increasing drug concentrations. LNP had hardly any effect on the reaction (not shown). Lansoprazole sulfide acted as a weak inhibitor of the reaction. At 100 μM, the cytochrome *c* reduction rate was decreased by ∼50% (Figure 4c). For comparison, antimycin A and atovaquone would be 1,000-fold more potent in the same conditions. We anticipate that AmB and yeast metabolism would help LNP transform into a more potent metabolite than both LNP and LNPS.

### Molecular docking supports the binding of LNP and its metabolites to cytochrome *bc_1_*

**Figure 5.**
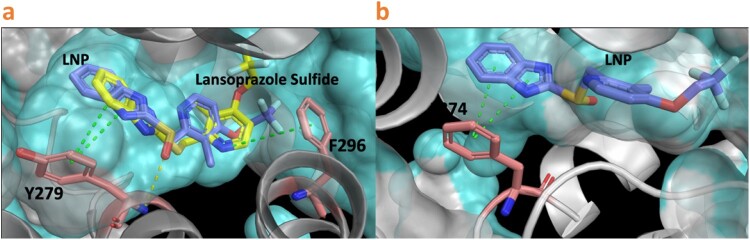
Docking analysis of *C. albicans* (PDB ID: 7RJA) and crystal structure analysis of Bovine *bc_1_* with UHDBT (PDB ID: 1SQV). a) Predicted binding mode of LNP and lansoprazole sulfide to the binding site of 7RJA. Green dashed line, pi-pi interaction, yellow dashed line, hydrogen bond. b) Predicted binding mode of lansoprazole to the binding site of 1SQV. Green dashed line, pi-pi interaction.

We determined the predicted docking poses for LNP and the lansoprazole sulfide to the *C. albicans* cytochrome *bc_1_* cryoEM structure (PDB: 7RJA). Based on the docking affinities determined by Glide (Schrödinger LLC), LNP shows a slightly more favourable pose with a Glide XP score of −10.8 kcal/mol (Figure 5a) while this score is −10.4 for Lansoprazole sulfide. Both LNP and Lansoprazole sulfide adopt similar extended poses, mainly stabilized by hydrophobic contacts in the pocket. Similar in both compounds, the imidazole ring forms a T-shape stacking with the side chain of Y279 (Figure 5a) and the pyridine ring forms pi-pi and halogen-pi interactions with F296. However, LNP has an additional hydrogen bond with the sulfide oxygen and the backbone amine of Y279, which may explain the different docking scores. Finally, our results also correlate with the experimental selectivity toward the *C. albicans* cytochrome *bc*_1_ (PDB: 7RJA), as the docking score to the bovine *bc1* with UHDBT (PDB ID: 1SQV) structure is –8.2 kcal/mol, which is 2.6 kcal/mol higher than that of the *C. albicans* cytochrome *bc*_1_ structure (Figure 5b). Other metabolites of LNP showed worse docking scores with the wild type and tested mutants (Table S6). Overall, our docking studies provide rational support for the association between LNP and its metabolic products and the binding to cytochrome *bc*_1_.

### The AmB/LNP combination has *in vivo* efficacy in a murine model of *C. auris* infection

**Figure 6.**
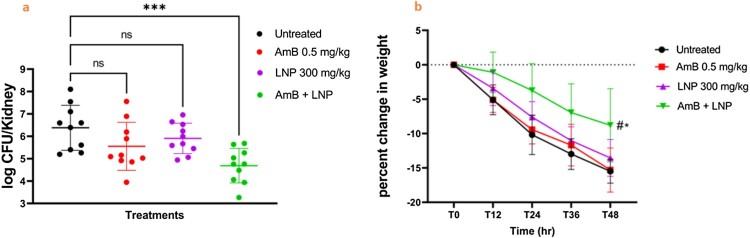
Lansoprazole (LNP) potentiates the antifungal activity of amphotericin B (AmB) in a murine model of *C. auris* infection. a) Groups of female CD-1 mice (10 mice per group) were infected with AmB–resistant *C. auris* AR0390 (2.6 × 10^7^ CFU/mouse) and treated with vehicle control (untreated), AmB (0.5 mg/kg), LNP 300 mg/kg, or a combination of both drugs. The burden of *C. auris* in murine kidneys (log CFU) was determined from a single experiment. A dot on the graph represents each mouse. The data were analyzed via a one-way analysis of variance (ANOVA) using post-hoc Dunnett’s test for multiple comparisons. The asterisks (***) indicate a statistically significant difference (*P* < 0.05) compared to the untreated control. b) Monitoring the weight of CD-1 mice in the murine model of *C. auris* infection. Percent changes in weight were calculated for 48 hr. Data are presented as mean +/- SE. The asterisk (*) and pound (#) signs indicate a statistically significant difference compared to the untreated and the AmB-treated cells, respectively, as determined via a two-way ANOVA using Dunnett’s test for multiple comparisons.

The therapeutic potential of the AmB/LNP combination was evaluated in a disseminated infection mouse model. First, we tested different therapeutic doses of AmB and identified the highest dose that does not cause a significant reduction in CFU as a single daily dose of 0.5 mg/kg for two days (Fig. S2). Then, we evaluated the efficacy of the AmB/LNP (0.5/300 mg/kg) combination in an established murine model of systemic *C. auris* infection [39]. LNP (300 mg/kg) significantly enhanced the activity of AmB (0.5 mg/kg) as the AmB/LNP combination reduced the fungal burden in the kidneys by 1.7-log (98%) reduction compared to untreated mice and by one log (90%) reduction compared to the AmB-treated group. The AmB/LNP combination resulted in reduced weight loss in treated mice, demonstrating the overall improved health of the mice (Figure 6a and 6b). The promising *in vivo* results of AmB/LNP combination provides an additional confirmation of the therapeutic potential of this combination in the treatment of *C. auris* infections.

## Discussion

*C. auris* represents a paradigm shift in *Candida* infections [53]. With high resistance to the last resort antifungal agent (AmB) reported, *C. auris* introduced us to a new era of multidrug-resistant fungi [54]. The potential of antifungal combination therapy to treat multidrug- and pandrug-resistant *C. auris* has been previously reported [12]. However, a recent study highlighted the alarming problem of pan-drug-resistant isolates that do not respond to combination therapy [55]. In this study, we screened 727 FDA-approved drugs and clinical molecules to identify drugs that can restore the antifungal activity of AmB against multidrug-resistant *C. auris*. LNP exhibited a synergistic relationship with AmB against 90% of the tested isolates and restored the fungicidal capability of AmB within 6 hr. Additionally, this combination was effective against other medically important fungal pathogens, including drug-resistant *C. glabrata* and *Cryptococcus* species.

The limited understanding of the mechanism of synergy is a key roadblock that hinders the clinical advancement of new therapeutic regimens. In this work, we explored the potential target of LNP, which helps to enhance the activity of AmB. We utilized a proteomics approach, the PISA assay, which assesses the alteration of the thermal stability of drug-bound proteins. LC-MS was performed to identify the thermal shift under different temperatures [30]. The PISA analysis identified eight potential targets for LNP, including the *C. auris* orthologs of *S. cerevisiae RIP1* and *TUB1*. We became interested in the mitochondrial cytochrome *bc_1_* due to its overlap with the secondary mode of action of AmB, which involves causing oxidative cell damage as evidenced by multiple studies [56]. Furthermore, a recent study showed that another cytochrome *bc_1_* inhibitor (Inz-5) alters the configuration of the Rieske head domain in mitochondrial cytochrome *bc_1_*, demonstrating the targetability of that complex [57]. Cytochrome *bc_1_* was also identified as a target for a LNP metabolite in *M. tuberculosis*, which further supported the hypothesis that cytochrome *bc_1_* is a potential target in *C. auris* [44].

To track the molecular response to AmB/LNP treatment, we performed RNA-Seq analysis for cells treated with each drug alone and in combination. The up and downregulated genes after treatment with either AmB (0.5 µg/mL) or AmB/LNP (0.5/30 µg/mL) were extensive and driven by DNA damage, which is consistent with oxidative stress that AmB places on *Candida* cells at these concentrations. In contrast, cells treated with LNP alone showed minimal effects, as only ten genes were affected. The common function between these genes collectively is a cellular response to oxidative stress.

**Figure 7.**
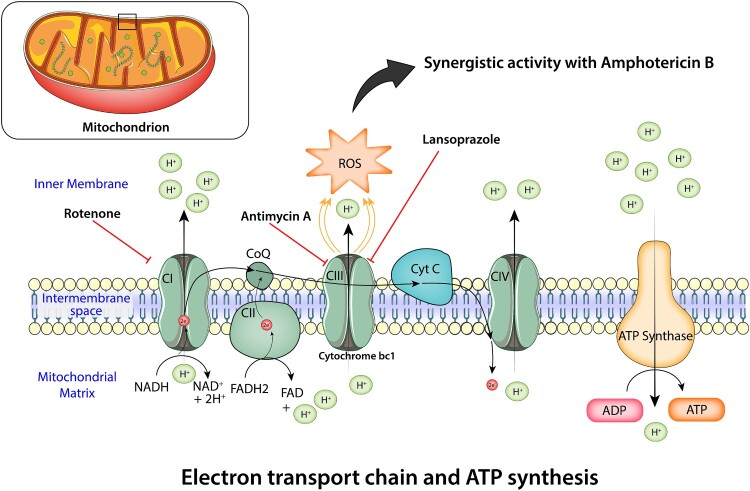
Diagram of the synergistic mechanism of the amphotericin B (AmB)/lansoprazole (LNP) combination. LNP/its metabolites inhibit fungal mitochondrial cytochrome *bc_1_* (complex III), leading to the generation of oxidative stress (reactive oxygen species [ROS]), and work synergistically with the antifungal activity of AmB.

The role of oxidative damage as a secondary mode of action for AmB has been highlighted in several reports [56]. Also, earlier studies pointed to a correlation between withstanding oxidative stress and AmB resistance in other fungal species [58]. As mentioned earlier, the evidence leads us to our central hypothesis that the fungal cytochrome *bc_1_* is the potential target by which LNP works synergistically with AmB (Figure 7).

Mitochondrial cytochrome *bc_1_* (complex III) inhibition is a major source of intracellular ROS generation [59]. Here, we showed that LNP generates ROS in *C. auris* and how the production of ROS considerably increased when LNP was combined with AmB. We also assessed the impact of standard respiratory complexes inhibitors (rotenone and antimycin A) on the AmB/LNP activity. In line with our hypothesis, rotenone and antimycin A enhanced the antifungal activity of AmB against *C. auris*. We confirmed that rotenone can improve the activity of AmB but also can block further synergy between AmB with either LNP or antimycin A. The combination of inhibitors strongly supports that in the presence of a high concentration of rotenone, the electron transport chain is disrupted at an earlier step (complex I), which prevents cytochrome *bc_1_* inhibitors, such as antimycin A, from generating more ROS. Consistent with our hypothesis, deleting the major ROS-scavengers, superoxide dismutases in *S. cerevisiae* led to a severe increase in yeast sensitivity to LNP.

The importance of the respiratory function for the activity of LNP was also supported by the decreased sensitivity of *S. cerevisiae* rho° mutant (lacking a respiratory chain) to LNP as compared to its parent strain and by the reduced sensitivity of *C. albicans* grown in glucose medium as compared to glycerol medium that favours respiration. It should be noted that despite the poor ability to grow on glycerol or ethanol as the carbon source [50], *C. auris* metabolism is likely to favour respiration because proteomic profiling demonstrated relative enrichment of tricarboxylic acid (TCA) cycle and decreased glycolysis proteins compared to *C. albicans* [60].

Using prepared mitochondria from *S. cerevisiae*, we observed that LNP had hardly any direct effect on cytochrome *bc*_1_ activity, while LNP metabolite lansoprazole sulfide (the active metabolite identified against *M. tuberculosis* [44]) acted as a (very) weak inhibitor of the enzymatic activity. Of note, we examined the synergy between lansoprazole sulfide and AmB, but it did not show any synergy against *C. auris*. LNP might target the fungal cytochrome *bc*_1_ through other metabolites (which may be aided by the AmB treatment). Molecular docking analysis showed that LNP and four metabolites could possibly bind to Q_o_ site of cytochrome *bc*_1_ and that small changes in the drug structure could alter its binding to the target.

The involvement of cytochrome *bc*_1_ in LNP-induced growth inhibition was further supported by the finding that in *S. cerevisiae*, the cytochrome *b* mutation Y279C resulted in an increased sensitivity to LNP in growth assays. Molecular docking of LNP and several known metabolites to Y279C mutant did not demonstrate greatly enhanced binding compared to the wild-type protein. The cause of this enhanced sensitivity is still to be understood and could be due to the action of unidentified metabolite(s).

The cytochrome *bc_1_* is a crucial component of cellular respiration [61] and it is unlikely that fungi can easily tolerate its inhibition. Previous research highlighted the importance of cytochrome *bc1* as a potential antifungal drug target [61,62]. Also, previous studies reported that targeting fungal cytochrome *bc_1_* disrupts the ability of *C. albicans* to tolerate exposure to antifungal drugs, sensitizes *C. albicans* to macrophages, and, most importantly, impairs the virulence of *C. albicans* in mice [62]. On the other hand, some inhibitors to cytochrome *bc_1_* were deemed to be toxic to human cells [63]. Even though cytochrome *bc_1_* exists in human mitochondria, other FDA-approved medications, like atovaquone, target plasmodial cytochrome *bc_1_* safely, which validates cytochrome *bc_1_* as a suitable drug target [64].

A crucial step in drug development is demonstrating *in vivo* efficacy because many compounds cannot disseminate through biological barriers and successfully cure an illness. The efficacy of the AmB/LNP combination in the murine model emphasizes the therapeutic potential of such combinations. Interestingly, the AmB/LNP combination was well tolerated, with no signs of toxicity observed in any of the treated mice, improving the therapeutic potential for this combination.

In conclusion, our study employed a drug repurposing strategy and identified lansoprazole as having a potent capability to restore and enhance the *in vitro* and *in vivo* antifungal activities of amphotericin B. This research highlights the importance of cytochrome *bc1* as a potential antifungal target, emphasizing its role in augmenting the antifungal effectiveness of amphotericin B particularly against drug-resistant *C. auris*. Furthermore, our data provides an additional support for the clinical potential of lansoprazole and other related compounds as a promising scaffold in overcoming resistance to amphotericin B in *Candida auris*.

## Limitations

One limitation of the effectiveness of the AmB/LNP combination against *C. auris* is the elevated doses of LNP required to demonstrate an *in vitro* and *in vivo* synergistic relationship with AmB. Additionally, LNP is an unstable compound, yielding several metabolites and further research is necessary to identify the exact active metabolite of LNP and elucidate its formation process. Furthermore, comprehensive toxicological studies in mice are required to assess the potential adverse effects of the combination. However, this work emphasized the role of cytochrome *bc1* as a potential antifungal target that enhances AmB's antifungal efficacy against drug-resistant *C. auris* isolates. Furthermore, we demonstrated the *in vivo* potential of using cytochrome *bc1* inhibitors in combination with AmB to treat a drug-resistant fungal infection.

## Supplementary Material

lansoprazole_supplemental_clean

Excel_sheet_S1

## Data Availability

All RNA-seq data are available in the NCBI database (Gene Expression Omnibus [GEO]) under the accession number GSE244094.
