# Supplementary material for: Lansoprazole interferes with fungal respiration and acts synergistically with amphotericin B against multidrug-resistant Candida auris
Source: Emerg Microbes Infect. 2024 Mar 3;13(1):2322649. doi: 10.1080/22221751.2024.2322649 (PMC10911247; doi:10.1080/22221751.2024.2322649)
Supplement: lansoprazole_supplemental_clean [file TEMI_A_2322649_SM0962.docx]

**Supplementary material**

**Lansoprazole interferes with fungal respiration and acts synergistically with amphotericin B against multidrug-resistant *Candida auris***

Ehab A. Salama^a,b^, Yehia Elgammal^a,b^, Aruna Wijeratne^c^, Nadia A. Lanman^d,e^, Sagar M. Utturkar^d^, Atena Farhangian^f^, Jianing Li^f^, Brigitte Meunier^g^, Tony R. Hazbun^d,f#^, Mohamed N. Seleem^a,b#^ *^a^ Department of Biomedical Sciences and Pathobiology, Virginia-Maryland College of Veterinary Medicine, Virginia Polytechnic Institute and State University, Blacksburg, Virginia, 24061, USA.*

*^b^ Center for One Health Research, Virginia Polytechnic Institute and State University, Blacksburg, Virginia, 24061, USA.*

*^c^ Department of Biochemistry and Molecular Biology, Indiana University School of Medicine, Indianapolis, Indiana, USA.*

*^d^ Purdue Institute for Cancer Research, Purdue University, West Lafayette, Indiana, USA.*

*^e^ Department of Comparative Pathobiology, Purdue University, West Lafayette, Indiana, USA.*

*^f^ Department of Medicinal Chemistry and Molecular Pharmacology, Purdue University, West Lafayette, IN 47907, USA.*

*^g^ Université Paris-Saclay, CEA, CNRS, Institute for Integrative Biology of the Cell (I2BC), 91198 Gif-sur-Yvette, France.*

**#Corresponding Authors:**

Tony R. Hazbun

Department of Medicinal Chemistry and Molecular Pharmacology,

Purdue University, West Lafayette, IN 47907, USA.

Hansen Life Sciences Research Building

201 S. University Street

West Lafayette, IN 47907-2064

765-496-8228, thazbun@purdue.edu

Mohamed N. Seleem

Department of Biomedical Sciences and Pathobiology

Virginia-Maryland College of Veterinary Medicine

Virginia Polytechnic Institute and State University

1410 Prices Fork Rd, Blacksburg, Virginia, 24061

Phone: 540-231-7173

Email: [seleem@vt.edu](mailto:seleem@vt.edu)

**Fungal isolates, media, chemicals, and tested drugs.**

A total of 34 fungal isolates were used in this study (20 *C. auris*, 2 *C. albicans*, 2 *C. glabrata*, 2 *C. tropicalis,* 2 *C. parapsilosis*, 2 *C. krusei,* 2 *C. neoformans*, and 2 *C. gattii*). Isolates were obtained from the CDC (Atlanta, GA, USA), BEI Resources (Manassas, VA, USA), and Westerdijk Fungal Biodiversity Institute (Utrecht, Netherlands).

References to the sources of the isolates along with susceptibility to antifungal agents are provided through the following links.

<https://wwwn.cdc.gov/arisolatebank/ARBCitations>

<https://www.beiresources.org/Catalog/Fungi/NR-52715.aspx>

<https://wi.knaw.nl/>

Other parent and mutant strains used in this study and their description are listed in Table S1. *S. cerevisiae* parent strain BY4741 and mutants were obtained from Horizon Discovery (Cambridge, United Kingdom). *S. cerevisiae* strain AD1-9 lacks several genes controlling drug uptake and export (*MAT*a *ura3 his1 yor1Δ::hisG snq2Δ::hisG pdr5Δ::hisG pdr10Δ::hisG pdr11Δ::hisG ycf1Δ::hisG pdr3Δ::hisG pdr15Δ::hisG pdr1Δ::hisG*) which renders the cell sensitive to drugs [1]. The strain was kindly provided by M. Ghislain, UCL, Belgium. The *Δsod1* and *Δsod2* mutants were constructed by PCR-based method [2]. The cytochrome *b* mutants were generated by the mitochondrial transformation technique [3]. The rho° mutant was generated by ethidium bromide treatment as in [4].

**Table S1: Effect of the combination of AmB and LNP against different *Candida* and *Cryptococcus* species.**

| **Isolate ID** | **MIC (µg/mL)** | | | | **ΣFICI** | **Interpretation** |
| --- | --- | --- | --- | --- | --- | --- |
|  | **Alone** | | **Combined** | |  |  |
|  | **AmB** | **LNP** | **AmB** | **LNP** |  |  |
| ***C. albicans* NR-29448** | 1 | >128 | 0.25 | 32 | 0.38 | SYN |
| ***C. albicans* ATCC 64124** | 1 | >128 | 0.25 | 32 | 0.38 | SYN |
| ***C. glabrata* 328** | 1 | >128 | 0.25 | 32 | 0.38 | SYN |
| ***C. glabrata* 581** | 1 | >128 | 0.25 | 16 | 0.31 | SYN |
| ***C. tropicalis* ATCC 1369** | 1 | >128 | 0.5 | 8 | 0.53 | IND |
| ***C. tropicalis* ATCC 13803** | 1 | >128 | 0.25 | 16 | 0.31 | SYN |
| ***C. parapsilosis* CAB 502638** | 0.5 | >128 | 0.125 | 16 | 0.31 | SYN |
| ***C. parapsilosis* ATCC 22019** | 0.5 | >128 | 0.125 | 16 | 0.31 | SYN |
| ***C. krusei* ATCC 34135** | 2 | >128 | 0.5 | 16 | 0.31 | SYN |
| ***C. krusei* CAB 396420** | 2 | >128 | 0.5 | 16 | 0.31 | SYN |
| ***C. gattii* NR-43210** | 1 | 128 | 0.25 | 32 | 0.50 | SYN |
| ***C. gattii* NR-43209** | 1 | 128 | 0.25 | 16 | 0.37 | SYN |
| ***C. neoformans* NR-41298** | 1 | 128 | 0.25 | 16 | 0.37 | SYN |
| ***C. neoformans* NR-41299** | 1 | 128 | 0.25 | 32 | 0.50 | SYN |

**AmB**: amphotericin B, **LNP**: lansoprazole, **ΣFICI**: fractional inhibitory concentration index, **SYN**: synergy, **IND**: indifference.

**Table S2: Potential targets from PISA analysis with comparison between PISA-deltaSm and Global-deltaSm (Excel spreadsheet_S1).**

| **NO.** | **Protein target Description** | ***C. auris* Gene Name** | ***C. albicans* Gene Name** | ***S. cerevisiae* Gene Name** | **PISA-deltaSm** | **Global-deltaSm** |
| --- | --- | --- | --- | --- | --- | --- |
| 1 | Aminotran_1_2 domain-containing protein | B9J08_004909 | CR_08640C_A | LCB1 | 2.52 | 0.86 |
| 2 | Hypothetical protein | B9J08_000464 | CR_07660C_A | SRP102 | 1.48 | 0.43 |
| 3 | Uncharacterized protein | B9J08_000560 | CR_09660W_A | YLR287C | 1.44 | - |
| 4 | Tubulin alpha chain | B9J08_004499 | TUB1 | TUB1 | 1.39 | 0.78 |
| 5 | O-acyltransferase | B9J08_002807 | ARE2 | ARE2 | 1.32 | 0.38 |
| 6 | Hypothetical protein | B9J08_005489 | CR_04230W_A | NRK1 | 1.26 | 0.13 |
| 7 | Cytochrome *bc*_1_ complex subunit Rieske, mitochondrial | B9J08_005505 | RIP1 | RIP1 | 1.21 | 0.78 |
| 8 | Hypothetical protein | B9J08_000180 | MET18 | MET18 | 0.83 | 0.15 |

**Table S3: List of *Saccharomyces cerevisiae* mutants used in the study.**

| **Group** | **ID** | **Gene name** | **Function** | **Pathway** |
| --- | --- | --- | --- | --- |
| Superoxide dismutase mutants | Δ*sod1* | *SOD1* | Cyt/mito Superoxide dismutase | Superoxide scavengers |
|  | Δ*sod2* | *SOD2* | mitochondrial Superoxide dismutase | Superoxide scavengers |
| Cytochrome *b* mutants | AD1-9 | **-** | Parent strain | Cytochrome *bc*_1_ (Electron transport chain) |
|  | F129L | *CYTB* | Electron transfer complex |  |
|  | L275F | *CYTB* |  |  |
|  | F278A | *CYTB* |  |  |
|  | Y279C | *CYTB* |  |  |
| Tubulin mutants | BY4741 | - | Parent strain |  |
|  | YOR026W | *BUB3* | cell cycle | Tubulin |
|  | YGL086W | *MAD1* | cell cycle |  |
|  | YJL030W | *MAD2* | cell cycle |  |
|  | YGR188C | *BUB1* | cell cycle |  |
|  | YMR055C | *BUB2* | cell cycle |  |

**Table S4: Effect of Lansoprazole, cytochrome *bc*_1_ inhibitor (antimycin A) and tubulin inhibitors (Benomyl and mebendazole) on *S. cerevisiae* tubulin mutants.**

| ***S. cerevisiae* ID.** | **Gene name** | **MICs (µg/ml)** | | | | **Function** |
| --- | --- | --- | --- | --- | --- | --- |
|  |  | **LNP** | **Benomyl** | **AA** | **Mebendazole** |  |
| BY4741 (parent) | _ | >128 | 32 | >8 | >128 | _ |
| YOR026W | BUB3 | >128 | 8 | >8 | 8 | Tubulin |
| YGL086W | MAD1 | >128 | 32 | >8 | 8 | Tubulin |
| YJL030W | MAD2 | >128 | 32 | >8 | 8 | Tubulin |
| YGR188C | BUB1 | >128 | 8 | >8 | 8 | Tubulin |
| YMR055C | BUB2 | >128 | 32 | >8 | 8 | Tubulin |

**LNP**: lansoprazole, **AA:** Antimycin A.

**Table S5: GO slim summary of differentially expressed genes between AmB/LNP and AmB treatments.**

| **No.** | **GO slim Term** | **CLUSTER_FREQUENCY** |
| --- | --- | --- |
| **1** | **Generation of precursor metabolites and energy** | 100.00% |
| **2** | **Cellular respiration** | 100.00% |
| **3** | **Transmembrane transport** | 72.73% |
| **4** | **Monoatomic ion transport** | 72.73% |
| **5** | **Mitochondrion organization** | 18.18% |

**Table S6: Molecular docking scores:**

| **Structure/ Ligand** | **7RJA_WT**  **kcal/mol** | **7RJA_Y279C**  **kcal/mol** | **7RJA_Y279S**  **kcal/mol** | **1SQV_WT**  **kcal/mol** |
| --- | --- | --- | --- | --- |
| **Lansoprazole** | -10.789 | -10.356 | -10.180 | -8.228 |
| **Lansoprazole Sulfide** | -10.385 | -10.323 | -9.157 | -5.003 |
| **Lansoprazole Sulfone** | -9.100 | -9.417 | -10.447 | -9.333 |
| **5-Hyddroxy Lansoprazole** | -9.006 | -8.630 | -9.673 | -8.497 |
| **5-Hdroxy Lansoprazole Sulfide** | -9.168 | -9.583 | -9.313 | -6.761 |


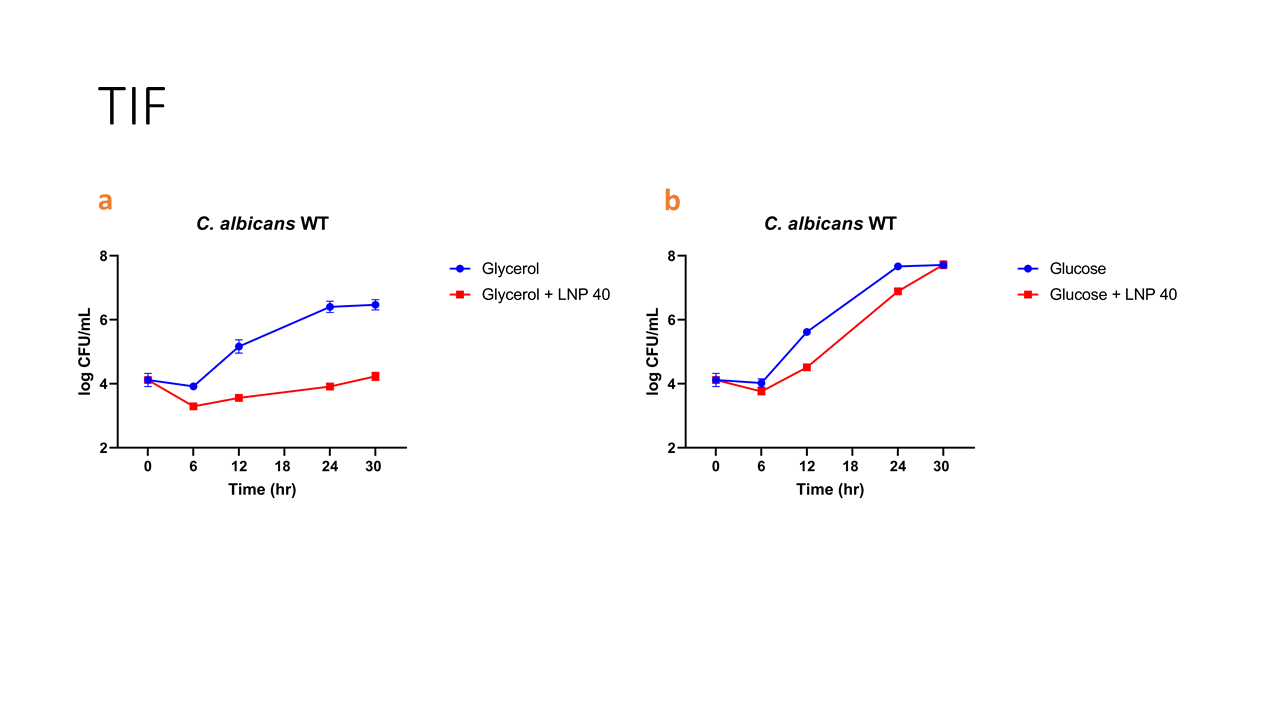


**Figure S1) LNP inhibits *C. albicans* growth when a) glycerol is the only carbon source and minimal effect was observed in b) glucose-enriched media.** *C. albicans* SC5314 was used in this experiment because it grows better on minimal media containing glycerol as a sole carbon source. The wild-type strain *C. albicans* was grown on YNB broth supplemented with glycerol (2%) or glucose (2 %) as a sole carbon source. The effect of LNP (40 µg/ml) on the growth (CFU) of *C. albicans* was determined in both media over a 30 hr incubation period at 35 °C. the data represents the average of two independent experiments.


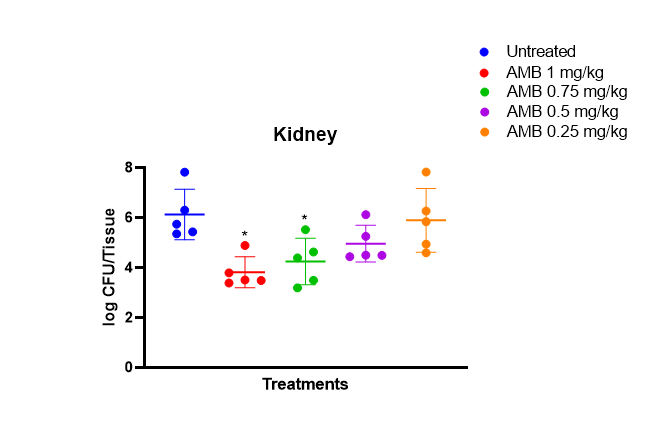


**Figure S2: *In vivo* optimization of AMB dose in murine model of *C. auris* infection.** Groups of female CD-1 mice (5 mice per group) were infected with AMB-resistant *C. auris* AR0390 (3.3 × 10^7^ CFU/mouse) and treated with vehicle control (untreated), or different dose of AMB (1,0.75, 0.5 and 0.25 mg/kg), daily for two days. On day three, mice were euthanized and the kidneys were extracted and the fungal burden was counted. The Kidney *C. auris* burden (log CFU) was determined and each mouse was represented by a dot in the graph. The data were analyzed via a one-way analysis of variance (ANOVA) using post-hoc Dunnett’s test for multiple comparisons. The asterisk (*) indicates a statistically significant difference (*P* < 0.05) compared to the untreated control.


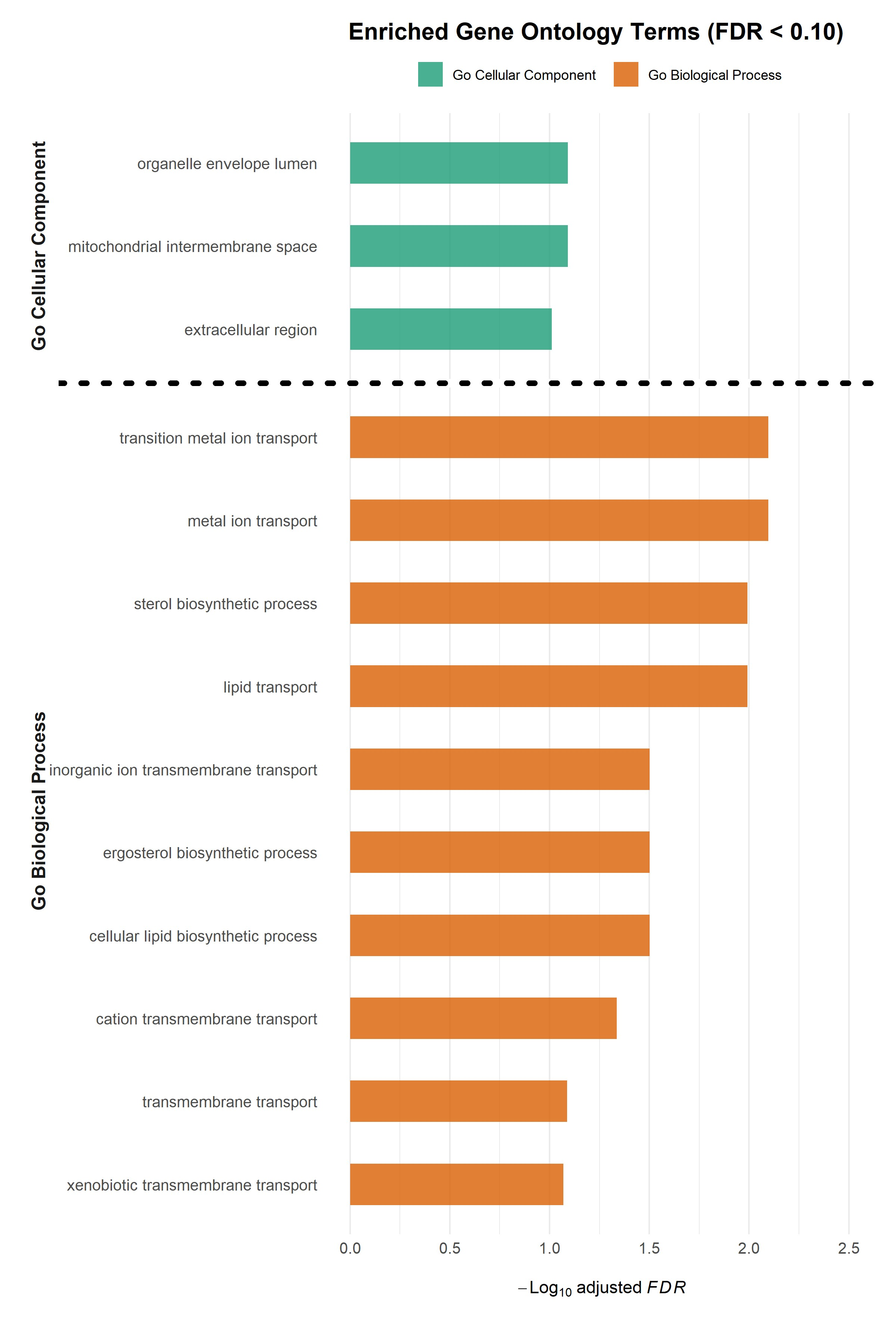


**Figure S3: Gene Ontology (GO) enrichment analysis.** GO enrichment analysis with differentially expressed genes from LNP-treated *C. auris* AR0390 highlights the *C. auris* mitochondrial intermembrane space as top Cellular Component (CC) location. GO enrichment analysis with differentially expressed genes from AmB/LMP combination-treated *C. auris* highlights “transport and biosynthetic processes” as top Biological Processes (BP).

**References**

1. Decottignies, A., et al., *ATPase and multidrug transport activities of the overexpressed yeast ABC protein Yor1p.* J Biol Chem, 1998. **273**(20): p. 12612-22.

2. Mounkoro, P., et al., *Investigating the mode of action of the redox-active antimalarial drug plasmodione using the yeast model.* Free Radic Biol Med, 2019. **141**: p. 269-278.

3. Hill, P., et al., *Recapitulation in Saccharomyces cerevisiae of cytochrome b mutations conferring resistance to atovaquone in Pneumocystis jiroveci.* Antimicrob Agents Chemother, 2003. **47**(9): p. 2725-31.

4. Laleve, A., et al., *The antimalarial drug primaquine targets Fe-S cluster proteins and yeast respiratory growth.* Redox Biol, 2016. **7**: p. 21-29.
